# Chromosome-level genome assembly of navel orange cv. Gannanzao (*Citrus sinensis* Osbeck cv. Gannanzao)

**DOI:** 10.1093/g3journal/jkad268

**Published:** 2023-11-24

**Authors:** Zhiwei Xiong, Hui Yin, Nian Wang, Guanzhu Han, Yuxia Gao

**Affiliations:** National Navel Orange Engineering Research Center, Gannan Normal University, Ganzhou, Jiangxi 341000, China; National Navel Orange Engineering Research Center, Gannan Normal University, Ganzhou, Jiangxi 341000, China; Citrus Research and Education Center, Department of Microbiology and Cell Science, IFAS, University of Florida, Lake Alfred, FL 33850, USA; College of Life Sciences, Nanjing Normal University, Jiangsu 210098, China; National Navel Orange Engineering Research Center, Gannan Normal University, Ganzhou, Jiangxi 341000, China

**Keywords:** navel orange cv. Gannanzao, navel orange cv. Newhall, genome, fruit ripening–related genes

## Abstract

Navel orange cv. Gannanzao is a variant of the navel orange cv. Newhall (*Citrus sinensis* Osbeck cv. Newhall) that exhibits an earlier maturation, making it commercially valuable. However, the mechanisms underlying its early maturation remain obscure. To address this question, we conducted genome sequencing and de novo assembly of navel orange cv. Gannanzao. The assembled genome sequence is 334.57 Mb in length with a GC content of 31.48%. It comprises 318 contigs (N50 = 3.23 Mb) and 187 scaffolds (N50 = 31.86 Mb). The Benchmarking Universal Single-Copy Orthologs test demonstrates 94.6% completeness. The annotation revealed 23,037 gene models, 164.95 Mb of repetitive sequences, and 2,554 noncoding RNAs. A comparative analysis identified 323 fruit ripening–related genes in navel orange cv. Gannanzao genome, while navel orange cv. Newhall genome contained 345 such genes. These genes were organized into 320 orthologous gene families, with 30.3% of them exhibiting differences in gene copy numbers between the 2 genomes. Additionally, we identified 15 fruit ripening–related genes that have undergone adaptive evolution, suggesting their potential role in advancing fruit maturation in navel orange cv. Gannanzao. Whole-genome sequencing and annotation of navel orange cv. Gannanzao provides a valuable resource to unravel the early maturation mechanism of citrus and enriches the genomic resources for citrus research.

## Introduction

Fruit ripening is a complex physiological and biochemical process that involves chlorophyll degradation, synthesis of flavonoids and carotenoids, aroma development, and fruit softening. Plant hormones, transcription factors, and DNA methylation play important roles in regulating this process (Bai *et al*. 2021; Li *et al*. 2021; Kapoor *et al*. 2022). When the fruit initiates ripening, developmental signals (e.g. sugars, NO, Ca^2+^) and environmental cues (e.g. light) lead to the accumulation of reactive oxygen species, triggering the synthesis and accumulation of abscisic acid (ABA) and concomitantly inhibiting the synthesis and action of gibberellins (GAs), indole-3-acetic acid (IAA), and cytokinins. They also synergistically promote the synthesis and action of ethylene, jasmonic acid (JA), salicylic acid, and brassinosteroids (BRs; Guo *et al*. 2018; Fernández-Milmanda *et al*. 2020; Li *et al*. 2021). Fruits can be categorized into 2 types: ethylene-dependent respiration climacteric fruits and ethylene-independent nonrespiration climacteric fruits (Pech *et al*. 2008). Hormones together constitute a complex regulatory network, which orchestrates the maturation of fruits with harmonious precision. The synergistic regulation of ABA, ethylene, and IAA is evident in nonrespiratory climacteric fruits (such as citrus). Both ABA–IAA interaction and ethylene–IAA interaction are observed in respiratory climacteric fruits, whereas ABA–ethylene interaction is found in both climacteric and nonclimacteric fruits. These interactions play critical regulatory roles during fruit ripening (Guo *et al*. 2018; Li *et al*. 2021; Qiao *et al*. 2021). In concert with hormonal regulation, transcription factors (e.g. NAC) collaborate with MYB as well as ethylene-related and ABA-related transcription factors to form a regulatory network that responds to endogenous and environmental signals, modulating different aspects of fruit ripening (Li *et al*. 2018; Cao *et al*. 2020; Kou *et al*. 2021; Martín-Pizarro *et al*. 2021).

Citrus fruits are the largest category of fruits in the world and the third-largest traded agricultural commodity globally, holding a significant position in international agricultural trade and serving as an economic pillar for many citrus-growing regions. However, the limited number of commercially viable citrus varieties and their concentrated ripening periods have hindered the development of the citrus industry. Breeding of early-maturing and late-maturing varieties is an important objective in citrus breeding. Navel orange cv. Gannanzao, derived from a bud mutation of navel orange cv. Newhall, is an early-maturing variety known for its early and abundant fruit set, strong resistance, excellent quality, and significant economic impact, making it one of the main cultivated varieties in Gannan, one of the largest naval orange-producing regions worldwide (Long *et al*. 2019). Navel orange cv. Gannanzao ripens at the end of September to early October, whereas other naval orange cultivars ripen around mid-to-late November (Liu *et al*. 2019). However, the mechanism of its early ripening remains obscure. Here, we sequenced and assembled the chromosome-level haploid genome of navel orange cv. Gannanzao. Additionally, we explored the early ripening mechanism by conducting a comparative genomic analysis of navel orange cvs. Gannanzao and Newhall.

## Materials and methods

### Specimen collection, DNA extraction, and sequencing

Navel orange cv. Gannanzao genome was sequenced in this study. Plant materials were collected from the National Navel Orange Engineering Research Center in Ganzhou, Jiangxi Province, China.

Genomic DNA extraction was performed using the phenol-chloroform method (Rana *et al*. 2019) and isolated using the Nanobind Plant Nuclei Big DNA Kit (Circulomics Inc., Baltimore, MD, USA), following the manufacturer's instructions. The Illumina library was prepared according to the manufacturer's protocol (Kozarewa *et al*. 2009) and sequenced using the Illumina NovaSeq 6000 platform, generating 150-bp paired-end reads. For PacBio sequencing, a HiFi SMRTbell Library was constructed using 15 kb DNA fragments, and sequencing was performed using the PacBio Sequel II platform. Hi-C libraries were constructed (Belton *et al*. 2012), which were used for subsequent sequencing with the Illumina HiSeq-2500 platform, generating 125-bp paired-end reads.

### De novo genome assembly and genome quality assessment

HiFi reads were assembled into scaffolds using Hifiasm v0.15.4 with the Overlap-Layout-Consensus algorithm (Cheng *et al*. 2021). Leveraging the Hi-C sequencing data, the scaffold sequences were then elevated to the near-chromosome level using the LAchesis software (Burton *et al*. 2013). To attain a chromosome-level genome, manual corrections were made based on the intensity of chromosome interactions, analyzed by using the juicebox v1.11.08 tool (Durand *et al*. 2016). The quality of the assembly was assessed by employing the Benchmarking Universal Single-Copy Orthologs (BUSCO) v5.4.7 (Manni *et al*. 2021) and the Conserved Core Eukaryotic Genes Mapping Approach (CEGMA) v2 (Parra *et al*. 2007). Furthermore, the coverage of the assembled genomes was determined by mapping Illumina short reads to the assembly using the Burrows-Wheeler Aligner (BWA; Li and Durbin 2010).

### Genome annotation

In our repeat annotation pipeline, we employed a comprehensive approach that combines homology alignment and de novo search to identify repeats throughout the entire genome. Tandem repeats were identified using TRF (Benson 1999) through ab initio prediction. For homolog prediction, the widely used Repbase database was utilized along with the RepeatMasker software (Tempel 2012) and its in-house scripts (RepeatProteinMask), using default parameters to extract repeat regions. In addition, a de novo database of repetitive elements was built using LTR_FINDER (Xu and Wang 2007), RepeatScout (Lian *et al*. 2015), and RepeatModeler (Flynn *et al*. 2020) with default parameters. The raw transposable element (TE) library was then constructed, including all repeat sequences with lengths >100 bp and gaps composed of “N” <5%. To identify DNA-level repeats, a custom library combining Repbase and the de novo TE library (processed using uclust to generate a nonredundant library) was provided to RepeatMasker.

To predict gene models, we employed a combination of homology-based prediction, ab initio prediction, and RNA-seq–assisted prediction. Homology-based prediction involved aligning genomic sequences to homologous proteins using tblastn v2.2.26 (Camacho *et al*. 2009) with a threshold of *E*-value ≤ 1*e*−5. Gene structure prediction was then performed using GeneWise v2.4.1 software (Birney 2004), based on the matched proteins from reference genomes. For automated de novo gene prediction, we utilized Augustus v3.2.3 (Nachtweide and Stanke 2019), Geneid v1.4 (Blanco *et al*. 2007), Genescan v1.0 (Burge and Karlin 1997), GlimmerHMM v3.04 (Majoros *et al*. 2004), and SNAP_2013-11-29. To annotate the genome, transcriptome assembly was conducted using Trinity v2.1.1 (Haas *et al*. 2013). For the identification of exon regions and splice positions, RNA-seq reads from leaf, root, and fruit tissues were aligned to the genome using Hisat v2.0.4 (Kim *et al*. 2019) with default parameters. The alignment results were then used as input for StringTie v1.3.3 (Pertea *et al*. 2015) with default parameters. Finally, a nonredundant reference gene set was generated by merging genes predicted from the 3 methods using EvidenceModeler (EVM) v1.1.1 (Haas *et al*. 2008).

Gene functions were assigned based on the protein sequences’ best match by aligning them with Swiss-Prot (Boutet *et al*. 2007) using Blastp (Camacho *et al*. 2009), with a threshold of *E*-value ≤ 1*e*−5. To annotate motifs and domains, InterProScan70 v5.31 (Quevillon *et al*. 2005) was employed to search against various publicly available databases such as ProDom (Servant *et al*. 2002), PRINTS (Attwood and Beck 1994), Pfam (Mistry *et al*. 2020), SMRT (Lou *et al*. 2020), PANTHER (Mi *et al*. 2019), and PROSITE (Hulo 2006). The corresponding Gene Ontology (Gene Ontology Consortium 2014) IDs for each gene were assigned according to the relevant InterPro (Hunter *et al*. 2009) entry.

For predicting protein function, we transferred annotations from the closest BLAST hit in the Swissprot20 (Boutet *et al*. 2007) database and DIAMOND v0.8.22 (Buchfink *et al*. 2014) with an E-value <10−5, as well as the NR database (Pruitt *et al*. 2007) with a similar E-value <10−5. Furthermore, we mapped the gene set to a KEGG pathway (Kanehisa *et al*. 2017) to identify the most suitable match for each gene.

### Synteny analysis

To investigate the synteny between navel orange cvs. Gannanzao and Newhall, we identified the orthologous genes using OrthoFinder v2.5.4 (Emms and Kelly 2019) with a default parameter. According to the structural annotation file (gff3), we extracted the positional in-genome and sequence length information orthologous genes. MCScanX software (Wang *et al*. 2012) with a default parameter was performed to complete the synteny analysis.

### Selection pressure analysis

Orthologous gene families were identified using OrthoFinder v2.5.4 (Emms and Kelly 2019) with a default parameter. Based on functional annotation, we extracted gene families containing genes related to fruit ripening. The nucleotide sequences of each gene family were aligned using the MEGA software and subsequently manually edited. The *Ka* (nonsynonymous substitution rate) and *Ks* (synonymous substitution rate) were calculated using the Nei–Gojobori method with the Jukes–Cantor substitution model implemented in DNASP 6 (Rozas *et al*. 2017). For multigene families, DNASP 6 (Rozas *et al*. 2017) calculates *Ka/Ks* for every 2 sequences and then computes the mean value.

## Results and discussion

### Genome sequencing and assembly

Navel orange cv. Gannanzao was sequenced using PacBio seq II for long-read sequencing, Illumina NovaSeq 6000 for shot-gun sequencing, and Illumina HiSeq-2500 for Hi-C, which produced 94 (∼267-fold coverage), 48, and 45 Gb data, respectively. Illumina and PacBio data were used for de novo assembly of the genome. The genome size of navel orange cv. Gannanzao was determined to be 334.67 Mb, with 318 contigs and an N50 value of 3.23 Mb (Table 1). Furthermore, there were 187 scaffolds with an N50 of 31.86 Mb. The GC content was 34.48%. With the assistance of Hi-C technology, the genome assembly of navel orange cv. Gannanzao was improved to chromosome level, resulting in 9 pairs of chromosomes. The haploid genome size was 302.61 Mb, accounting for 90.42% of the genome (Supplementary Table 1). Additionally, 178 scaffolds (32.06 Mb, 9.58%) could not be assembled to chromosome level. The Hi-C heatmap illustrates a robust intrachromosomal interaction within the same chromosome, indicating a high-level chromosomal condensation quality in the genome of navel orange cv. Gannanzao (Supplementary Fig. 1).

**Table 1.** Genome assembly and annotation information of Gannanzao navel orange and Newhall navel orange.

Term	Navel orange cv. Gannanzao	Navel orange cv. Newhall
Chromosome number (2*n*)	18	18
Total size of genome (Mb)	334.57	325.89
Number of contigs	318	183
ContigN50 (Mb)	3.23	4.04
Number of scaffolds	187	102
Scaffolds N50 (Mb)	31.86	31.52
GC content	34.48	34.37
Repeat elements (Mb/%)	164.95/48.29	153.07/46.95
Number of gene models	23,037	24,504
Average transcript length (bp)	3,125.38	3,082.49
Average CDS length (bp)	1,198.99	1,177.05
Average exons per gene	5.24	5.14
Average exon length (bp)	228.78	229.21
Average intron length (bp)	454.26	460.78
Number of ncRNA		
miRNA	554	529
tRNA	431	451
rRNA	100	290
snRNA	1,469	1,420
BUSCO (%) *n* = 1,440		
Complete	94.6	95.5
Complete and single copy	84.2	86.3
Complete duplicated	10.4	9.2
Fragmented	1.2	1.1
Missing	4.2	3.4
CEGMA CEGs = 248		
Gene number	232	230
Completeness (%)	93.55	92.74
BWA		
Mapping rate of reads (%)	91.65	94.46
Coverage of genome (%)	99.94	99.97

The completeness of the navel orange cv. Gannanzao genome assembly was evaluated using BUSCO (Manni *et al*. 2021). The BUSCO completeness was estimated to be 94.6% (Table 1), indicating a relatively complete genome assembly. The completeness of the navel orange cv. Gannanzao genome assembly was also evaluated using CEGMA (Parra *et al*. 2007). This approach utilizes 248 Core Eukaryotic Genes (CEGs) found in 6 eukaryotic model organisms to construct a core gene set. The evaluation revealed that, of the 248 CEGs, 232 were successfully assembled, accounting for a completeness rate of 93.55% (Table 1). This result further supports that the genome assembly of navel orange cv. Gannanzao is relatively complete. To evaluate the accuracy of the genome assembly, a small fragment library of reads was selected and aligned to the assembled genome using BWA software (Li and Durbin 2010). Approximately 91.65% of the small fragment reads were aligned with the genome assembly, and the coverage rate was 99.94% (Table 1). These results demonstrate a high level of consistency between the reads and the assembled genome. In summary, the genome assembly of navel orange cv. Gannanzao was evaluated using multiple methods, and the results showed high consistency, completeness, and accuracy of the genome.

We compared the navel orange cv. Gannanzao genome sequence with that of navel orange cv. Newhall (Table 1). The 2 genomes have similar characteristics, such as genome size, GC content, and values of N50 for contigs and scaffolds (Table 1). Taken together, all the data indicate that the genome assembly of navel orange cv. Gannanzao is of high quality.

### Genome annotation

Repeat sequences in the navel orange cv. Gannanzao genome were identified using a combination of de novo prediction and homology-based alignment. The repeat sequences account for 49.29% of the genome (Table 1). The repeat sequences were classified into 2 types: tandem repeat and interspersed repeat. Through annotation, we found that the tandem repeat sequences consist of 14,446,157 bp, accounting for 4.32%. Meanwhile, the dispersed repeat sequences consist of 150,499,547 bp (44.97%) of the genome, with long terminal repeat retrotransposons being the most abundant components, accounting for 127,474,950 bp (38.09%; Supplementary Fig. 2).

Gene structure annotation revealed a total of 23,037 genes in the navel orange cv. Gannanzao genome, with an average CDS length of 1,199 bp (Table 1). Each gene contains an average of 5.24 exons, and the average lengths of the exons and introns are 228.79 and 454.26, respectively (Table 1). A comparative analysis of navel orange cv. Gannanzao and 3 other closely related species (please list the names here; Xu *et al*. 2012; Wu *et al*. 2014; Gao *et al*. 2023) demonstrates that navel orange cv. Gannanzao exhibits a notable degree of similarity to the closely related species. This finding supported the high quality of the genome structure annotation of navel orange cv. Gannanzao (Supplementary Fig. 3).

Alignment of the predicted protein sequences obtained from gene structure annotation with a known protein database revealed that 96.8% of the genes could be functionally annotated (Supplementary Fig. 4 and Supplementary Table 2). Non-coding RNA (ncRNA) refers to RNA that does not code for proteins, such as rRNA, tRNA, snRNA, and miRNA. These RNAs play important biological functions. Through comparison with a known ncRNA database, 554 miRNAs, 431 tRNAs, 100 rRNAs, and 1,469 snRNAs were identified in the navel orange cv. Gannanzao genome (Table 1).

### Comparative genomic analysis with navel orange cv. Newhall

**Fig. 1.**
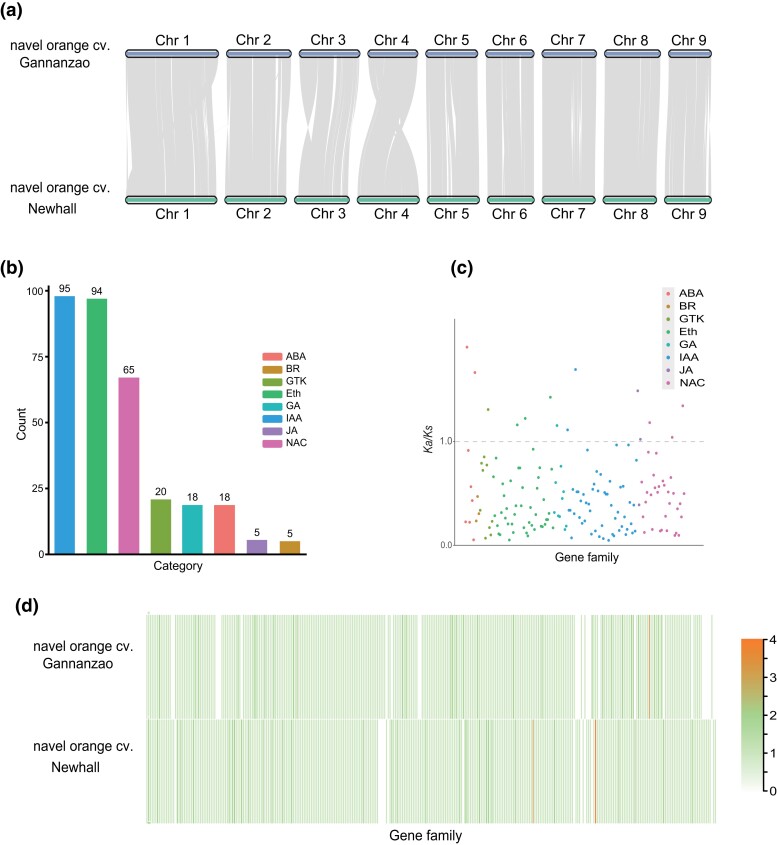
Comparative genomic analysis of navel orange cvs. Gannanzao and Newhall. a) Chromosome synteny plot of 2 citrus genomes. b) Number of fruit ripening–related genes. c) *Ka/Ks* value of fruit ripening–related genes. The dashed line represents the *Ka/Ks* ratio of 1. d) The copy number of the gene families of fruit ripening–related genes in 2 genomes.

We conducted a chromosome synteny analysis between navel orange cv. Gannanzao and its parent variety navel orange cv. Newhall. A similar pattern was observed between the 2 genomes (Fig. 1a). However, significant chromosomal translocation and inversions were observed. Next, we focused on the early ripening mechanism of navel orange cv. Gannanzao. Based on the annotation information of both genomes, we identified 323 and 345 fruit ripening–related genes in navel orange cvs. Gannanzao and Newhall, respectively. Using the OrthoFinder software (Emms and Kelly 2019), 320 fruit ripening–related gene families (95 IAA, 94 ethylene, 65 NAC, 20 GTK, 18 ABA, 18 GA, 5 JA, and 5 BR) were identified (Fig. 1b). A statistical analysis of the copy numbers of fruit ripening–related genes in each gene family revealed that 223 gene families (69.7%) had similar copy numbers in both genomes. Compared with navel orange cv. Newhall, navel orange cv. Gannanzao exhibited a decrease in gene copy numbers in 40 gene families (12.5%) and an increase in gene copy numbers in 57 gene families (17.8%; Fig. 1d). An analysis of the selective pressure on all fruit ripening–related genes showed that 14 gene families had *Ka/Ks* > 1(Fig. 1c). These 14 gene families, including 15 ripening-related genes in navel orange cv. Gannanzao (1 ABA, 1 GTK, 3 ethylene, 2 GA, 3 IAA, 2 JA, 3 NAC), had likely undergone adaptive evolution, possibly playing a role in the early ripening mechanism of navel orange cv. Gannanzao.

## Supplementary Material

jkad268_Supplementary_Data

## Data Availability

The raw sequencing data of navel orange cvs. Gannanzao and Newhall have been deposited in the NCBI databases under BioProject accessions PRJNA997102 and PRJNA810206. The genome assemblies of navel orange cvs. Gannanzao and Newhall have been deposited in the NCBI databases under GenBank accession: JAUONY000000000 and JAUONX000000000. The genome annotation is available on the Zenodo data repository under accession doi: 10.5281/zenodo.8174988. The scripts of genome assembly and data analysis have uploaded at GitHub (https://github.com/Gannanzao-resource/Gannanzao-resource). Supplemental material available at G3 online.
