# Supplementary material for: Chromosome-level genome assembly of navel orange cv. Gannanzao (Citrus sinensis Osbeck cv. Gannanzao)
Source: G3 (Bethesda). 2023 Nov 24;14(2):jkad268. doi: 10.1093/g3journal/jkad268 (PMC10849316; doi:10.1093/g3journal/jkad268)
Supplement: jkad268_Supplementary_Data [file jkad268_supplementary_data.zip › Supplemental_Figures_G3-2023-404623.pdf]

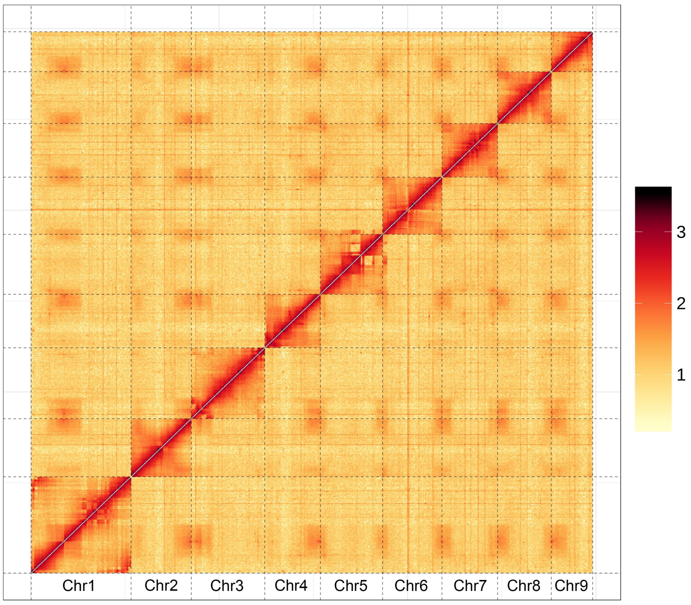

Figure S1. The heatmap of Hi-C

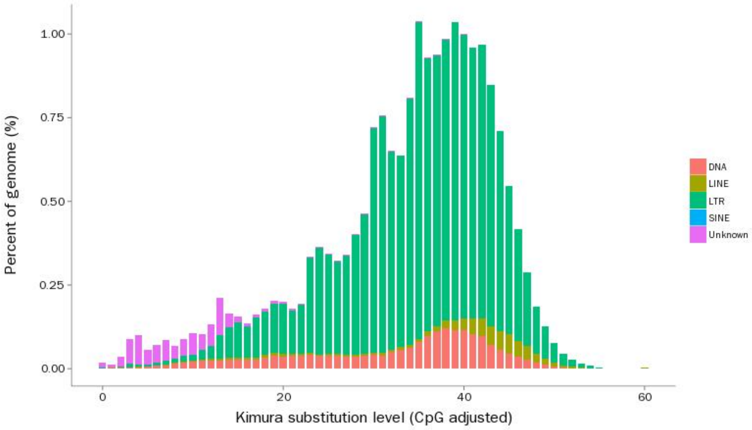

Figure S2. Distribution plot of the divergence degree of the TE

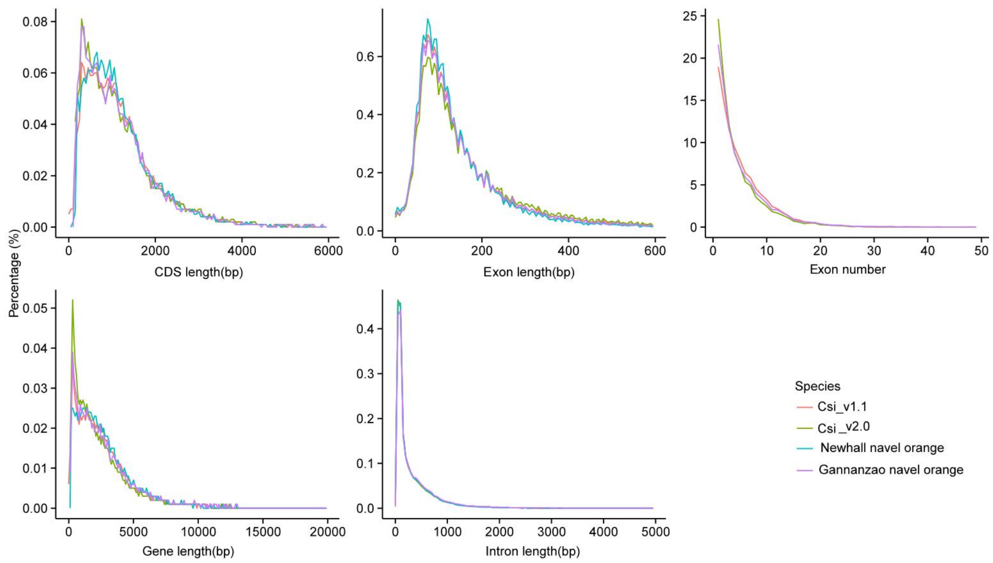

Figure S3. Comparative diagram of each element of a near-origin species

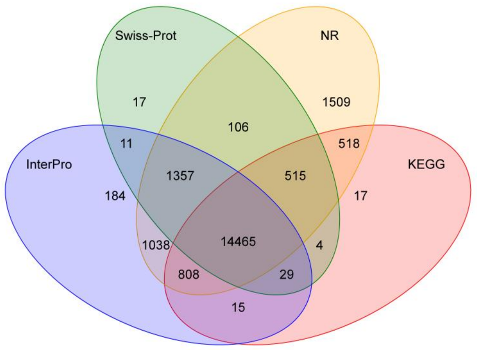

Figure S4. Statistical results of gene function annotation
