# Supplementary material for: Chromosome-level genome assembly of navel orange cv. Gannanzao (Citrus sinensis Osbeck cv. Gannanzao)
Source: G3 (Bethesda). 2023 Nov 24;14(2):jkad268. doi: 10.1093/g3journal/jkad268 (PMC10849316; doi:10.1093/g3journal/jkad268)
Supplement: jkad268_Supplementary_Data [file jkad268_supplementary_data.zip › Supplemental_Tables_G3-2023-404623.docx]

**Table S1. The length and cluster number for each chromosome in navel orange cv. Gannanzao genome**

| **Chromosome ID** | **Cluster Number** | **Sequence Length (bp)** |
| --- | --- | --- |
| Chr1 | 28 | 53,679,636 |
| Chr2 | 14 | 32,108,355 |
| Chr3 | 25 | 39,654,002 |
| Chr4 | 10 | 30,062,476 |
| Chr5 | 16 | 33,479,353 |
| Chr6 | 9 | 31,863,774 |
| Chr7 | 12 | 30,071,288 |
| Chr8 | 5 | 28,855,617 |
| Chr9 | 7 | 22,535,785 |
|  |  |  |

**Table S2. The statistics of gene functional annotation in navel orange cv. Gannanzao genome**

| **Item** | **Number** | **Percentage (%)** |  |
| --- | --- | --- | --- |
| Total | 23,037 | -- |  |
| Swissprot | 17,778 | 77.20 |  |
| Nr | 22,262 | 96.60 |  |
| KEGG | 17,283 | 75.00 |  |
| InterPro | 20,867 | 90.60 |  |
| GO | 12,723 | 55.20 |  |
| Pfam | 17,432 | 75.70 |  |
| Annotated | 22,292 | 95.80 |  |
| Unannotated | 745 | 3.20 |  |
